# Health-related quality of life in children with congenital heart disease aged 5 to 7 years: a multicentre controlled cross-sectional study

**DOI:** 10.1186/s12955-020-01615-6

**Published:** 2020-11-12

**Authors:** Hamouda Abassi, Helena Huguet, Marie-Christine Picot, Marie Vincenti, Sophie Guillaumont, Annie Auer, Oscar Werner, Gregoire De La Villeon, Kathleen Lavastre, Arthur Gavotto, Pascal Auquier, Pascal Amedro

**Affiliations:** 1 Paediatric and Congenital Cardiology Department, Arnaud De Villeneuve University Hospital, 371 Avenue du Doyen Giraud, 34295 Montpellier, France; 2 Paediatric Cardiology and Rehabilitation Unit, St-Pierre Institute, Palavas-Les-Flots, France; 3 Centre for Studies and Research On Health Services and Quality of Life, Public Health and Chronic Diseases Laboratory, Aix Marseille University, Marseille, France; 4 Epidemiology Department, University Hospital, Clinical Investigation Centre, INSERM–CIC 1411, University of Montpellier, Montpellier, France; 5 PhyMedExp, CNRS, INSERM, University of Montpellier, Montpellier, France

**Keywords:** Health-related quality of life, Patients-reported outcomes, Young children, Congenital heart disease

## Abstract

**Background:**

In the context of tremendous progress in congenital cardiology, more attention has been given to patient-related outcomes, especially in assessing health-related quality of life (HRQoL) of patients with congenital heart diseases (CHD). However, most studies have mainly focused on teenagers or adults and currently, few HRQoL controlled data is available in young children. This study aimed to evaluate HRQoL of children with CHD aged 5 to 7 y.o., in comparison with contemporary peers recruited in school, as well as the factors associated with HRQoL in this population.

**Methods:**

This multicentre controlled prospective cross-sectional study included 124 children with a CHD (mean age = 6.0 ± 0.8 y, 45% female) during their outpatient visit and 125 controls (mean age = 6.2 ± 0.8 y, 54% female) recruited at school. A generic paediatric HRQoL instrument was used (PedsQL 4.0).

**Results:**

Self-reported HRQoL in children with CHD was similar to controls, overall (73.5 ± 1.2 vs. 72.8 ± 1.2, P = 0.7, respectively), and for each dimension. Parents-reported HRQoL was significantly lower in the CHD group than in controls. HRQoL was predicted by the disease severity and by repeated invasive cardiac procedures (surgery or catheterization).

**Conclusion:**

HRQoL in young children with CHD aged 5 to 7 years old was good and similar to controls. This study contributed to the growing body of knowledge on HRQoL in congenital cardiology and emphasized the need for child and family support in the most complex CHD.

*Trial registration* This study was approved by the institutional review board of Montpellier University Hospital (2019_IRB-MTP_02-19) on 22 February 2019 and was registered on ClinicalTrials.gov (NCT03931096) on 30 April 2019, https://clinicaltrials.gov/ct2/show/NCT03931096.

## Background

In the past two decades, great advances in medical and surgical treatments for patients with congenital heart diseases (CHD) have significantly increased their life expectancy, and currently most children with CHD are expected to reach adulthood without any severe physical impairment (1, 2). Consequently, patients, healthcare professionals and researchers in paediatric cardiology have shown a growing interest in patient related outcomes (PROs), such as health-related quality of life (HRQoL) (3).

Previous studies have reported that many children with CHD were concerned with behavioural, emotional, or cognitive issues (4, 5). Moreover, some of these children may be trapped in the vicious circle of muscular deconditioning and therefore suffer from reduced exercise capacity, resulting in a lower HRQoL, despite the absence of any severe physical impairment (2, 6). In the current era, children with CHD should not be limited in any submaximal exercise performance (7) and we recently showed from a large cohort of nearly 800 children that most of them had a normal physical capacity, as measured by their maximal oxygen uptake (VO2_max_) (2). Nevertheless, parental overprotection and restriction recommendations by physicians have built some barriers to physical activity for youngsters with CHD (8–10).

In the literature, the existing correlation between HRQoL and the severity of the CHD has mostly been found when considering the physical dimension of HRQoL (6, 11, 12). Nevertheless, many patients develop a process of resilience including coping, response shift and sense of coherence, and therefore may have a good HRQoL, especially in the psychosocial dimensions (10, 13). As a result, depending on which aspect of HRQoL is concerned, some studies have reported a better HRQoL in children with CHD than in the general population (14), while other studies have mainly focused on the impact of the CHD severity on children’s HRQoL, and therefore reported an impaired quality of life (10, 15–17).

Nevertheless, most HRQoL studies with a consistent level of evidence have mainly included teenagers or adults with CHD, and, currently, few HRQoL controlled data is available in young children with CHD, especially in those under the age of 8 years (18, 19). Yet, many questions emerge during this period of life regarding young CHD children’s physical, emotional, and social well-being. Moreover, the transition from home to school requires developing new skills, involving physical activity, socialisation, autonomy, and self-confidence. As a result, this period may represent new challenges for young children with CHD, as well as for their families.

In this multicentre prospective controlled study, we aimed to evaluate HRQoL of children with CHD aged 5 to 7, in comparison with contemporary peers recruited in schools. We also intended to evaluate the factors associated with HRQoL in this population.

## Methods

### Study design

This multicentre controlled prospective cross-sectional study was carried out in two paediatric cardiology tertiary care centres (Montpellier University Hospital, France and St-Pierre Institute, Palavas-Les-Flots, France), and in 6 school classes in the Occitanie region, France, randomly selected from the Department of Education’s database.

### Patient’s population

Children with a CHD, as defined by the ACC-CHD classification (20), and aged 5 to 7 years old, were prospectively recruited in both centres during a paediatric cardiology outpatient visit. Inclusion procedures were beforehand harmonized. We did not include children with any other severe chronic disease (polymalformative genetic syndrome, extra-cardiac organ failure) and children unable to understand the questionnaire (e.g. non-French speakers, severe neurodevelopmental disorder). Children with any recent surgical or interventional cardiac catheterization procedures (6 monthsbeforeinclusionvisit) and hospitalized children were also excluded. Children filled-in their HRQoL self-questionnaire under trained clinical research assistant supervising, and their parents filled in the HRQoL proxy-questionnaire in a separate room, as in our similar previous studies (10, 18, 21, 22).

### Control population

In the 6 selected school classes, parents or legal guardians of all children aged 5 to 7 years were offered to participate in the study, with a common recruitment procedure for each class and similar to the one at the hospital. Children completed the HRQoL self-questionnaire under clinical research assistant supervising, at school. Parents filled in separately the HRQoL proxy-questionnaire at home.

### HRQoL questionnaires

We used the self and proxy versions of the PedsQL questionnaire dedicated to children aged 5 to 7 years. The PedsQL instrument is a generic paediatric HRQoL instrument, designed for children aged from 2 to 18 years, developed from a large cohort of healthy children as well as children with acute or chronic health conditions (23). The PedsQL questionnaire has four multidimensional scales: physical functioning (8 items), emotional functioning (5 items), social functioning (5 items), and school functioning (5 items). The three summary scores are the total scale score (23 items), the physical health summary score (8 items), and the psychosocial health summary score (15 items). Each item uses a 5-point Likert scale from 0 to 4 (0, never a problem; 1, almost never a problem; 2, sometimes a problem; 3, often a problem; 4, almost always a problem). For the 5–7 years self-report questionnaire, the Likert scale is reworded and simplified into a 3-point scale (0, not a problem; 2, sometimes a problem; 4, a lot of a problem). Items are reversed scored and linearly transformed to a 0–100 scale, higher scores indicating a better HRQoL. This instrument was validated by Varni et al. in healthy and patient populations and its psychometric properties showed reliability, validity and responsiveness to clinical change over time (23–25). The translation and cultural adaptation into French was performed by MAPI Research Institute (www.mapi-trust.org), following the international guidelines (26). The psychometric properties of the French version of the PedsQL appeared to be acceptable (27). We previously showed the good sensitivity of the PedsQL from several controlled prospective HRQoL studies among healthy controls and children with various chronic diseases (21, 28).

### CHD severity

The ACC-CHD classification was used to define the type of malformation (20). The disease severity was assessed by a paediatric cardiologist who was blinded to HRQoL reports. Three classifications were used to define CHD severity: (1) The Ross classification, using four severity groups (from Ross class I to Ross class IV) to grade the severity of congestive heart failure in infants (29); (2) the Bethesda classification, stratifying CHD in three severity categories (mild, moderate, and severe) (30); (3) and the prenatal prognostic scale from Davey et al. grading foetal cardiovascular diseases in 7 levels, from level 1 (cardiovascular finding with minimal, if any, negative impact on well-being), to level 7 (cardiovascular abnormality and complex form of CHD with very poor prognosis) (31).

### Clinical outcomes

In the CHD group, the following clinical variables were collected: gender, age, age at CHD diagnosis (pre or postnatal diagnosis), number of cardiac surgeries, number of interventional cardiac catheterization procedures, drug medication, and electrocardiographic status (presence of pacemaker or implantable defibrillator), and echocardiography data (left ventricle ejection fraction, right ventricle hypertension, pulmonary arterial hypertension, and mechanical valve).

In the control group, only gender and age were collected.

### Statistical analysis

The characteristics of the population were presented using mean and standard deviation for continuous variables, and frequencies and proportions for qualitative variables.

Age- and gender-adjusted means (± standard error of the mean, SEM) of HRQoL scores were measured (total, summary, and for each dimension). To compare HRQoL scores between CHD and controls with adjustment on age and gender, a covariance analysis (ANCOVA) was performed. The same method was used to study the effect of Ross classification (class I, class II, class III, class IV and controls) and Bethesda classification (mild severity, moderate severity, severe severity and controls) on HRQoL scores. For pairwise comparisons, the Holm’s correction was taken into account.

To identify the clinical factors associated with HRQoL summary scores (total, physical and psychosocial score) in the CHD group, a multiple linear regression was used. Except for age and gender, which were forced in the models, the other clinically relevant variables with a P value ≤ 0.20 in the univariate analysis were included in the model. The final model was obtained using a backward selection with a removal level of 0.10. Colinearity between factors was tested with variance inflation factors. To test the validity of the model, the normality of residues was tested with the Shapiro–Wilk test. The statistical significance was set at 0.05 and analyses were performed with SAS V.9 software.

## Results

### Population characteristics

A total of 249 children aged 5 to 7 years were recruited (mean age 6.1 ± 0.8 years, 49% female), including 124 subjects in the CHD group (mean age 6.0 ± 0.8 years, 45% female), and 125 subjects in the control group (mean age 6.2 ± 0.8 years, 54% female). Both groups were similar in terms of age and gender.

In the CHD group, the anomalies of the ventricular outflow tracts represented the most frequent sub-group (n = 44, 36%). In greater detail, the most frequent types of CHD were in the following order: ventricular septal defects (n = 32, 26%), anomalies of the atria and interatrial communications (n = 16, 13%), aortic valve stenosis (n = 14, 11%), and tetralogy of Fallot (n = 12, 10%). The smallest samples were complex anomalies of the atrioventricular connections (n = 2) and heterotaxy (n = 2). Most children in the CHD group had no congestive heart failure (Ross class I, 91%), nearly half of them had a low severity status (Bethesda mild severity, 47%), and nearly half of them had a good prognosis (Davey prognosis classification level 2, 48%). CHD diagnosis was performed prenatally in nearly one out of three cases. A total of 58 subjects (47%) underwent at least one cardiac surgical procedure and 29 subjects (24%) underwent at least one interventional cardiac catheterization procedure. Most patients had no cardiac medication and a normal left ventricular systolic function. Clinical characteristics of the CHD group were detailed in Table 1.

**Table 1:** CHD children’ medical characteristics

Variables			N (%)
ACC-CHD classification	1	Heterotaxy	2 (2)
	2	Anomalies of venous return	5 (4)
	3	Anomalies of the atria and interatrial communications	16 (13)
	4	Anomalies of the atrioventricular junctions and valves	6 (5)
	5	Complex anomalies of the atrioventricular connections	2 (2)
	6	Functionally univentricular hearts	3 (2)
	7	Ventricular septal defects (VSD)	32 (26)
	8.1	Transposition of the great arteries	7 (6)
	8.3	Tetralogy of fallot	12 (10)
	8.4	Anomalies of the intra-pericardial arterial trunks	3 (2)
	8.5	Aortic valve stenosis. Shone syndrome	14 (11)
	8.6	Pulmonary valve stenosis	8 (6)
	9	Anomalies of the extra-pericardial arterial trunks	11 (9)
	10	Congenital anomalies of the coronary arteries	3 (2)
Bethesda severity classification	Low		58 (47)
	Moderate		49 (40)
	Severe		17 (13)
Ross classification	Ross class I		112 (91)
	Ross class II		11 (9)
	Ross class III		0 (0)
	Ross class IV		0 (0)
Prognostic classification	Level 1		3 (2)
	Level 2		59 (48)
	Level 3		24 (19)
	Level 4		28 (23)
	Level 5		8 (6)
	Level 6		2 (2)
	Level 7		0 (0)
Age at CHD diagnosis	Prenatal		36 (32)
	Postnatal		75 (68)
Cardiac surgery	Yes		58 (47)
	No		65 (52)
Number of cardiac surgical procedures	1		47 (81)
	2		9 (16)
	≥ 3		2 (3)
Cardiac catheter	Yes		29 (24)
	No		94 (76)
Number of cardiac catheter procedures	1		25 (86)
	2		4 (14)
Cardiac device	Pacemaker or implantable defibrillator		1 (1)
	Mechanical valve		0 (0)
Cardiac treatment	Yes		16 (13)
	No		108 (87)
Pulmonary arterial hypertension	Yes		4 (3)
	No		119 (97)
Right ventricular hypertension	Yes		2 (2)
	No		121 (98)
Normal left ventricle ejection fraction	Yes		95 (93)
	No		7 (7)

Values are N (%)

*ACC-CHD* anatomic and clinical classification of congenital heart disease, *CHD* congenital heart disease

### HRQoL in children with CHD compared to control children

**Fig. 1:**
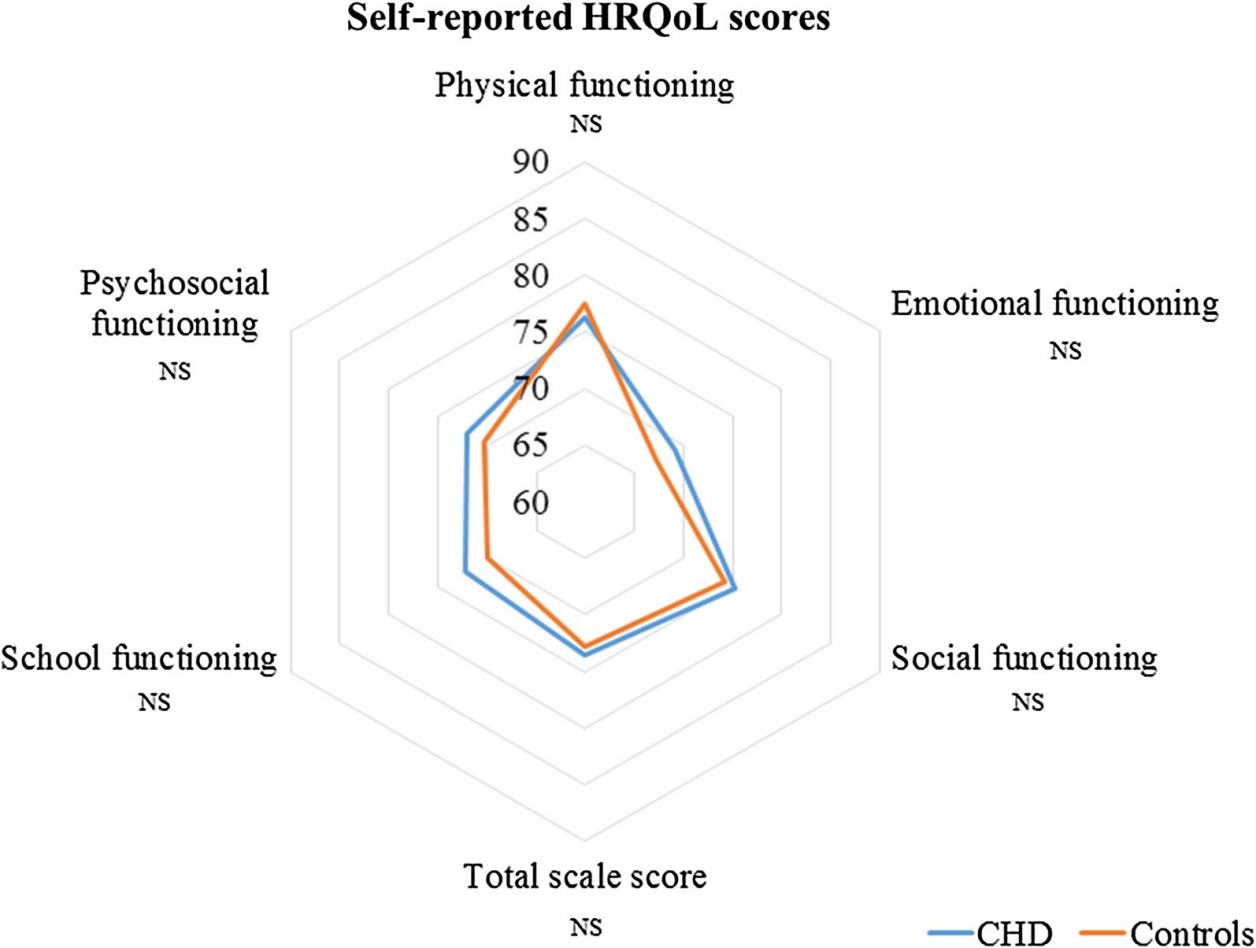
Self-reported HRQoL in children with CHD compared to control children. *HRQoL* health-related quality of life, *NS* non-significant

**Fig. 2:**
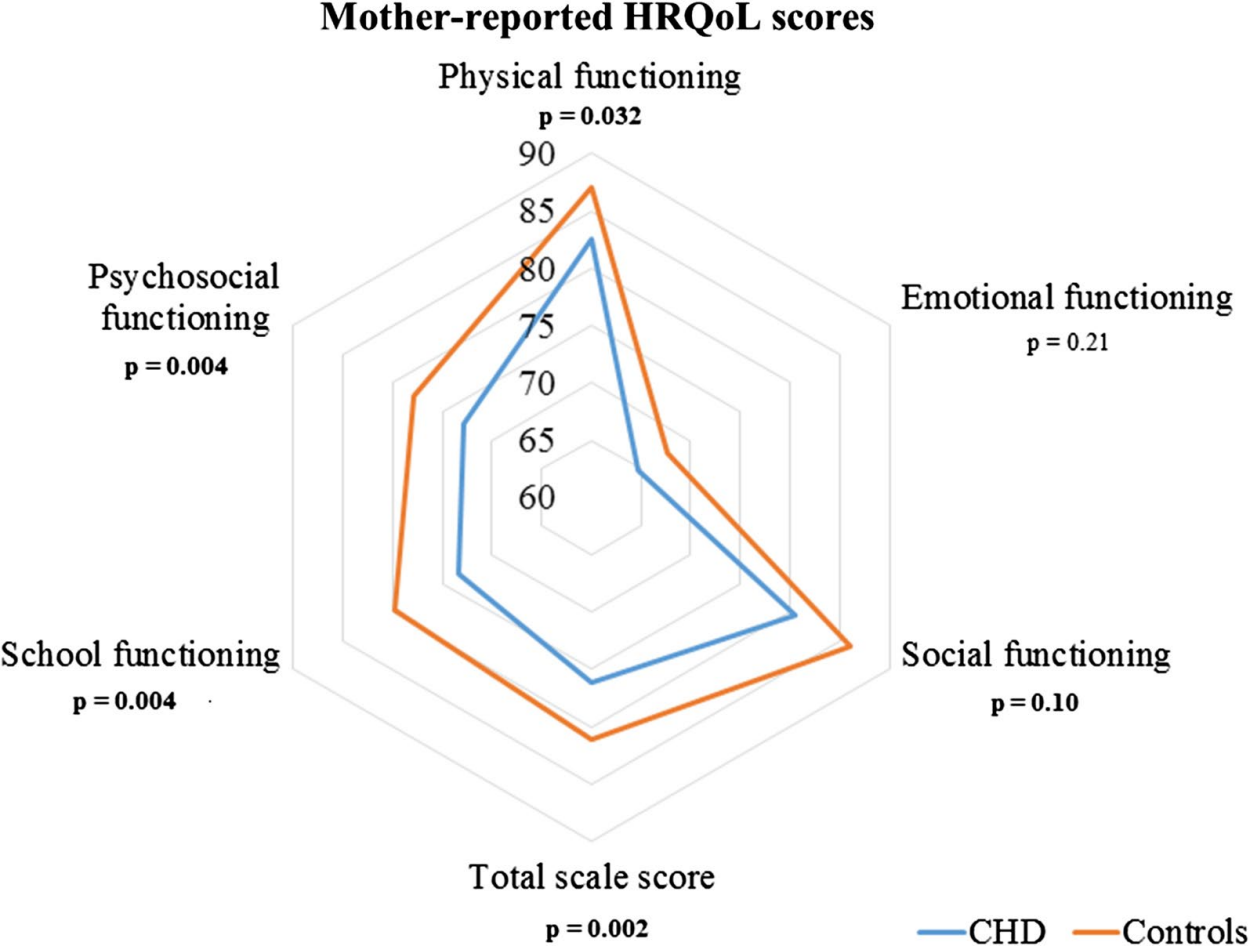
Mother-reported HRQoL in children with CHD compared to control children. *HRQoL* health-related quality of life, *NS* non-significant

**Fig. 3:**
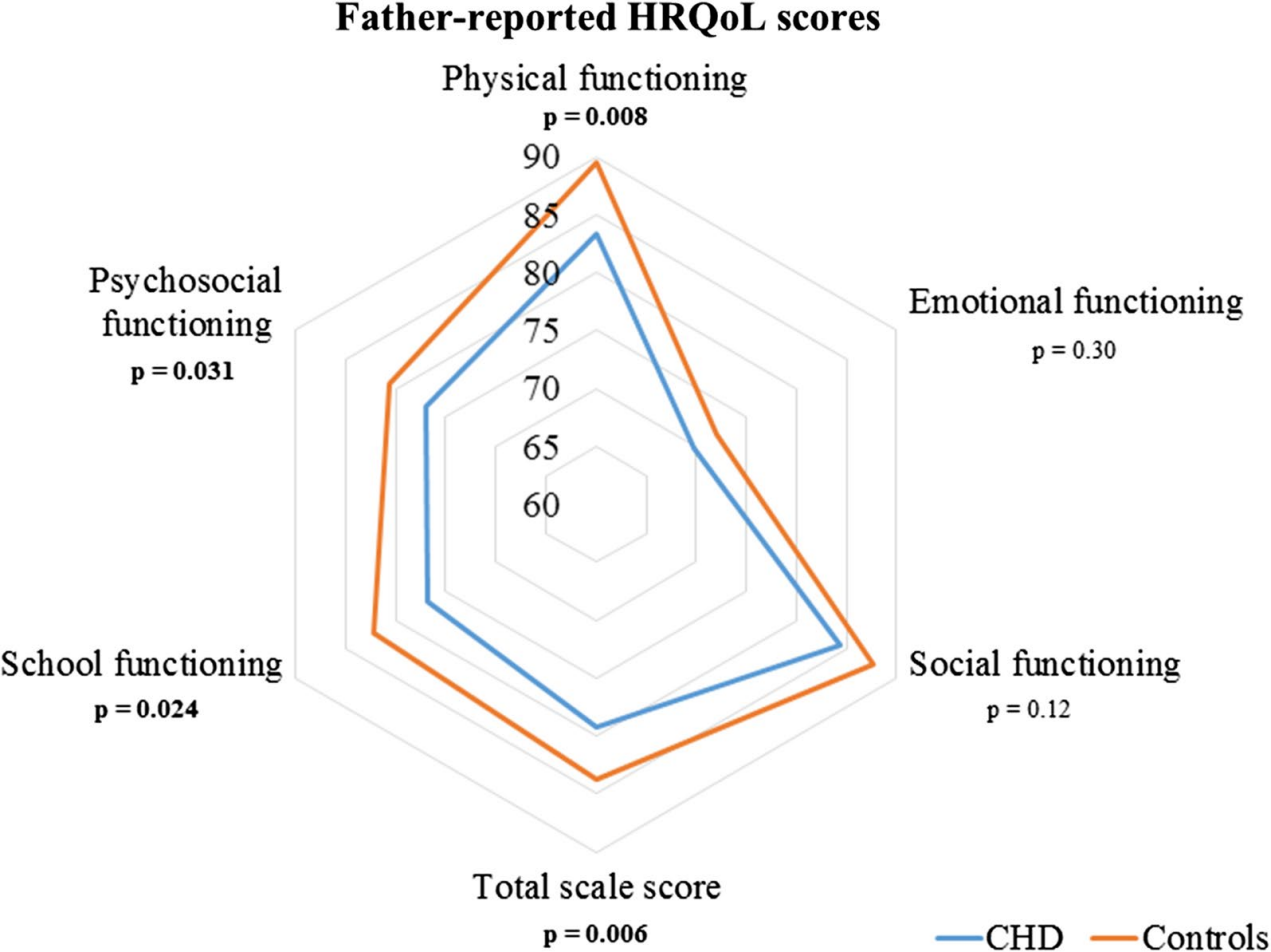
Father-reported HRQoL in children with CHD compared to control children. *HRQoL* health-related quality of life, *NS* non-significant

No significant differences were founded between CHD and control groups in self-reported HRQoL total score (73.5 ± 1.2 vs. 72.8 ± 1.2, P = 0.7, respectively) as in all dimensions (Fig. 1 and Additional file 1: Table 1). Mother-reported HRQoL was lower in the CHD group than in controls for total score (76.1 ± 1.1 vs. 81.1 ± 1.1, P = 0.002, respectively), and in all dimensions (P < 0.05) except for emotional functioning (Fig. 2 and Additional file 1: Table 1). Father-reported HRQoL was lower in the CHD group than in controls for total score (79.2 ± 1.2 vs. 83.7 ± 1.1, P = 0.006, respectively), physical functioning, school functioning, and psychosocial summary functioning (Fig. 3 and Additional file 1: Table 1).

### Relation between HRQoL and CHD severity

**Fig. 4:**
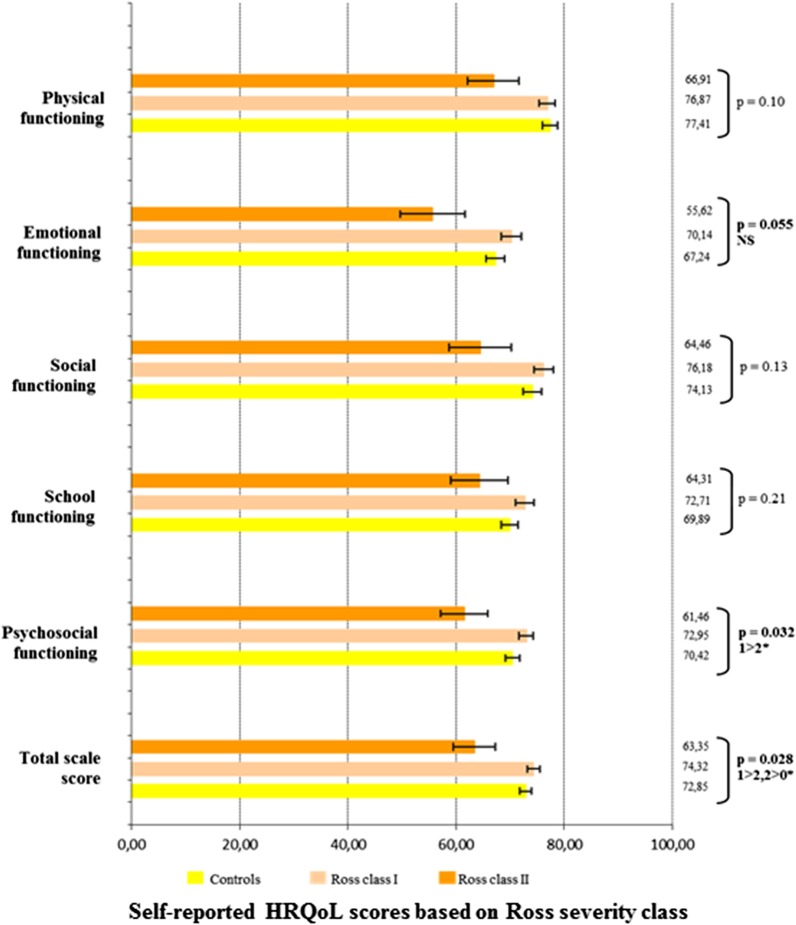
Self-reported HRQoL scores in the CHD group based on Ross severity class. *HRQoL* health-related quality of life. *0: Controls, 1: Ross class I, 2: Ross class 2

**Fig. 5:**
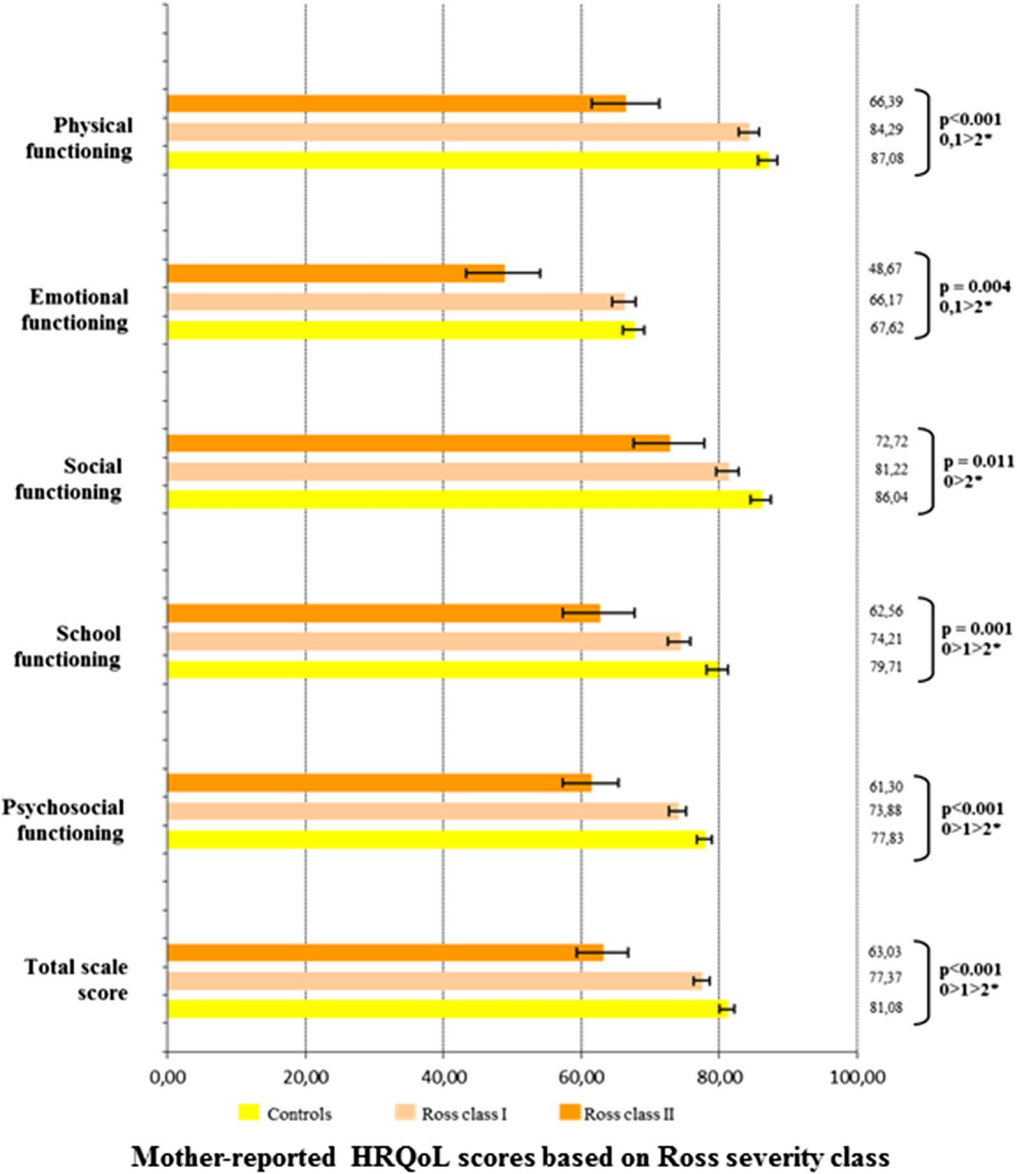
Mother-reported HRQoL scores in the CHD group based on Ross severity class. *HRQoL* health-related quality of life. *0: Controls, 1: Ross class I, 2: Ross class 2

**Fig. 6:**
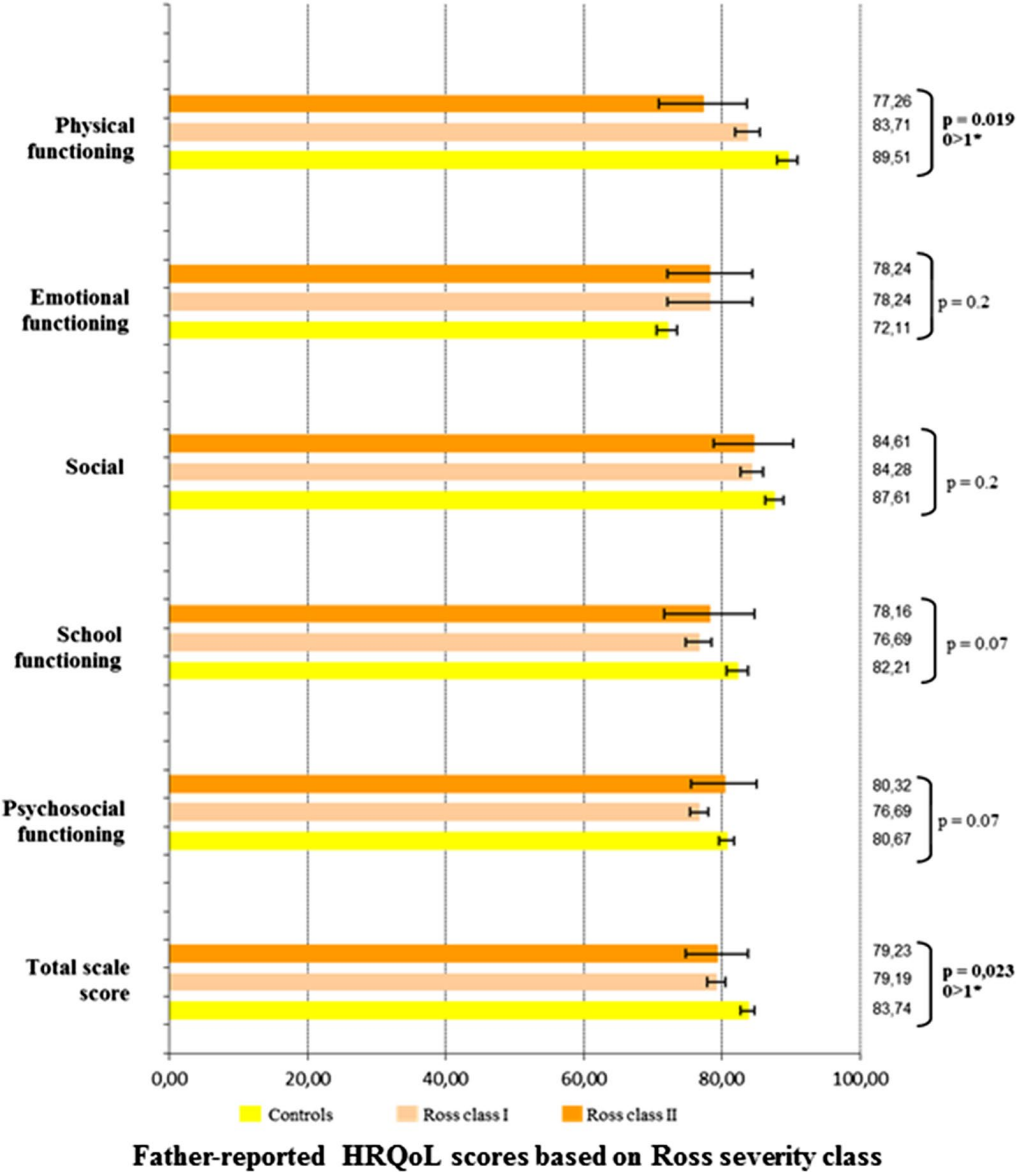
Father-reported HRQoL scores in the CHD group based on Ross severity class. *HRQoL* health-related quality of life. *0: Controls, 1: Ross class I, 2: Ross class 2

In terms of CHD severity, as assessed by the Ross classification, HRQoL analyses were performed between three groups of severity including the control group (controls, Ross class I and Ross class II), as no patient included in the study presented with Ross class III or IV status. Self-reported HRQoL was statistically different between those three severity groups, overall (controls, 72.9 ± 1.2; Ross I, 74.3 ± 1.2; Ross II, 63.4 ± 3.9; P = 0.03), in emotional functioning (P = 0.05), and in psychosocial summary functioning (P = 0.03) (Fig. 4). Mother-reported HRQoL scores were different between those three severity groups, overall (controls, 81.1 ± 1.1; Ross class I, 77.4 ± 1.2; Ross class II, 63.0 ± 3.8; P < 0.0001), as well as in all other dimensions (physical functioning, P < 0.0001; emotional functioning, P < 0.01; social functioning, P = 0.01; school functioning, P < 0.01; psychosocial functioning, P < 0.0001) (Fig. 5). Father-reported HRQoL was significantly different between the three severity groups, overall (controls, 83.7 ± 1.1; Ross class I, 79.2 ± 1.3; Ross class II, 79.2 ± 4.5; P = 0.02) and in physical functioning (P = 0.02) (Fig. 6). In the CHD group, the self- and mother-reported HRQoL scores (overall, physical health summary and psychosocial health summary) were affected by Ross classification (in univariate and multivariate analyses). The father reported HRQoL scores were not affected by this classification (Table 2, Additional file 1: Tables 2 and 3).

**Table 2:** Self-report QoL explanatory variables in the CHD group

Variables		Physical functioning			Psychosocial functioning			Total scale score		
		Univariate analysis		Multivariate analysis	Univariate analysis		Multivariate analysis	Univariate analysis		Multivariate analysis
		Estimation (SE)	p-value	p-value^$^	Estimation (SE)	p-value	p-value^$^	Estimation (SE)	p-value	p-value^$^
Age		0.3 (1.8)	0.89 *	0.72	− 1.6 (1.6)	0.31 *	0.46	− 1.0 (1.4)	0.50 *	0.93
Gender	Female versus male	− 4.3 (3.1)	0.16 *	0.07	− 0.4 (2.7)	0.88 *	0.34	− 1.8 (2.4)	0.46 *	0.17
Bethesda severity classification	Low versus severe	3.9 (4.7)	0.54	–	9.2 (4.0)	0.05 *	–	7.4 (3.6)	0.08 *	–
	moderate versus severe	0.7 (4.8)			4.4 (4.1)			3.1 (3.7)		
Ross classification	Class I versus Class II	9.2 (5.3)	0.08 *	*0.05*	11.2 (4.6)	0.02 *	*0.04*	10.5 (4.1)	0.01 *	*0.03*
Age at CHD diagnosis	Postnatal versus prenatal	3.9 (3.5)	0.26	–	4.6 (3.1)	0.13 *	–	4.4 (2.7)	0.11 *	–
Cardiac surgery	No versus yes	5.9 (3.0)	*0.05*	–	9.9 (2.5)	< *0.001*	–	8.5 (2.3)	< *0.001*	–
Number of cardiac surgical procedures	0 versus ≥ 3	6.9 (12.0)	0.10 *	–	16.9 (9.9)	< 0.001 *	< *0.01*	13.4 (9.0)	< 0.001 *	< *0.01*
	1 versus ≥ 3	2.7 (12.1)			9.2 (10.0)			6.9 (9.0)		
	2 versus ≥ 3	− 7.3 (13.1)			− 3.0 (10.8)			− 4.5 (9.8)		
Cardiac catheter	No versus yes	− 0.4 (3.6)	0.92	–	5.0 (3.1)	0.11 *	–	3.2 (2.8)	0.27	–
Number of cardiac catheter procedures	0 versus 2	− 6.9 (8.7)	0.71	–	1.8 (7.6)	0.25	–	− 1.2 (6.8)	0.42	–
	1 versus 2	− 7.5 (9.2)			− 3.8 (8.0)			− 5.1 (7.2)		
Cardiac medication	No versus yes	6.8 (4.5)	0.14 *	–	7.5 (3.9)	0.06 *	–	7.2 (3.5)	0.04 *	–
PAH	No versus yes	− 3.8 (8.6)	0.66	–	4.6 (7.6)	0.54	–	1.7 (6.8)	0.80	–
Normal left ventricle ejection fraction	No versus yes	− 2.1 (6.9)	0.76	–	− 0.1 (5.8)	0.98	–	− 0.8 (5.3)	0.88	–

Values are linear regression coefficients β (Standard Error) and p-values. Significant p-values are marked in italic

*CHD* congenital heart disease, *PAH* pulmonary arterial hypertension, *SD* standard error

^*^Candidate variables for multivariate analysis

^$^ “–” Not maintained in the model (P > 0.10)

In terms of CHD severity, as assessed by the Bethesda classification, self-reported HRQoL scores comparisons were significant between the four severity classes (controls, mild severity, moderate severity, and severe severity) in social functioning (P = 0.03) and psychosocial functioning (P = 0.05). However, no differences were found in the total scale score (controls, 72.9 ± 1.2; mild severity, 76.4 ± 1.7; moderate severity, 71.8 ± 1.9; severe severity, 68.6 ± 3.2; P = 0.1). Mother-reported HRQoL was different between the four severity groups, overall (controls, 81.1 ± 1.1; mild, 77.9 ± 1.7; moderate, 75.2 ± 1.8; severe, 72.9 ± 3.1; P = 0.01), as well as in physical functioning (P = 0.02), school functioning (P = 0.01), and psychosocial functioning (P = 0.02). Father-reported HRQoL scores comparisons were different between the four classes, overall (controls, 83.7 ± 1.1; mild, 79.5 ± 1.8; moderate, 79.8 ± 2.0; severe, 76.5 ± 3.2; P = 0.04), and in physical functioning (P = 0.05). Among CHD patients, Bethesda classification univariate and multivariate analyses found no association with self and proxy-HRQoL scores (Table 2 and Additional file 1: Tables 2 and 3).

Effect assessment of CHD severity on HRQoL according to the prenatal prognostic scale from Davey et al. could not be performed, given the small number of subjects in each one of the 7 scales.

### Relation between HRQoL and clinical outcomes in children with CHD

When considering the three PedsQL summary scores (e.g. total scale score, physical health summary score, and psychosocial health summary score), self-reported HRQoL was affected by the number of cardiac surgical procedures in the total scale score and in the psychosocial functioning (Table 2). Mother-reported HRQoL scores were affected by the administration of a cardiac medication on the physical, psychosocial and total scores (Additional file 1:Table 2).

Father-reported HRQoL was affected by the presence and the number of cardiac surgical procedures in psychosocial functioning, and in the total scale score, in the univariate analysis (Additional file 1: Table 3).

HRQoL was not significantly affected by the remaining clinical outcomes, such as the moment when the diagnosis of CHD was performed (prenatal vs. postnatal diagnosis), or the presence of a pulmonary arterial hypertension, or an impaired left ventricular function (Table 2, Additional file 1: Tables 2, 3).

## Discussion

This multicentre prospective controlled study of a cohort of 249 children aged 5 to 7 years old assessed HRQoL of 124 children with CHD in comparison with 125 controls. This study also evaluated the relation between CHD severity and HRQoL and investigated some clinical determinants of HRQoL in young children with CHD.

This study highlighted that self-reported HRQoL in young children with CHD was good, with a total PedsQL score of 73.5 ± 1.2 (out of 100), and therefore similar to that of their contemporary peers recruited in schools (e.g. 72.8 ± 1.2). Such HRQoL levels probably reflect all tremendous medical advances in the screening and care of CHD in the last decades, in terms of prenatal diagnosis, non-invasive real-time imaging, neonatal cardiac surgery, paediatric intensive care, and cardiac catheterization. Indeed, in the current era, over 90% of children with a CHD are expected to survive more than 30 years after first cardiac surgery (1). In our study, 91% of children with CHD had no symptoms of heart failure (Ross class I). As a result, more attention has been recently given to patient related outcomes in the field of congenital cardiology, beyond the classical cardiac morbidity and mortality outcomes (32, 33). Yet, more consistent HRQoL data from controlled studies are necessary in young children with CHD. Nevertheless, our results are in line with the study from Pilla et al., reporting that psychosocial domain of self-reported HRQoL was similar between children with CHD and healthy children aged from 3 to 7 years old (34). Conversely, the single-centre controlled study from Uzark et al. concluded that HRQoL in children aged 2 to 17 y.o. in the USA was significantly impaired, however the 5–7 y.o. age group represented the smallest sample of the study (17). From a general perspective, French healthcare system, as in most European countries, provides full coverage from the social security for patients with CHD, which is not the case in the USA. Therefore, we should be aware that HRQoL in chronic diseases also reflects the quality of a health care system, beyond the medical care itself (35, 36). Moreover, modern medical care strategies in paediatric cardiology involve educational programs, shorter hospital stays, and home-based healthcare programs, which may contribute to achieve good HRQoL levels (19, 21, 37). Classically, patients with CHD are prone to cope with their health condition, and develop adaptive skills such as reponse shift (38) or sense of coherence (13, 39), which may result in good HRQoL levels, especially in the psychological domains (18). This adaptation has been particularly shown in non-progressive health conditions, such as cerebral palsy, or in children with a correctable congenital anomaly, such as CHD, but less so for children with a progressive disease (40). To our knolewdge, adaptation processes have not been fully investigated yet in young children with CHD, but our results may also suggest an early process of “resilience”.

When focusing on proxy reports, our study found that parents-reported HRQoL was significantly lower in the CHD group than in controls, with an approximate mean difference of 5 points (out of 100) in both mother and father reports. Our results are consistent with previous similar studies, but few HRQoL data involving both mothers and fathers of the same child have been reported in paediatric cardiology (15, 17, 18, 18, 21, 28, 41). Nevertheless, parents-reported HRQoL scores in our study ranged from 74 to 84 points (out of 100), which is good and remains higher than scores reported in previous studies (21). We may hypothesize that leaving home and attending kindergarten or the first years of elementary school may be a challenge for parents of children with a CHD (42). Indeed, our results found lower proxy-reported HRQoL levels in school and psychosocial domains. Parents of children with CHD classically overprotect them and may fear that their children will be stigmatized, bullied or put on the sideline at school (18, 32, 41, 43). In general, parents of children with chronic health diseases are prone to developp stress and anxiety, undermining the well-being or overall stability of their family, as well as their ability to cope (44–46). In paediatric cardiology, factors contributing to parental stress may involve a past trauma, as after prenatal diagnosis, an anguish of death during cardiac surgery, and an inability to empower their child (47). Therefore, it is necessary to integrate psychosocial support dedicated to such parents (17, 48) and develop multidisciplinary family-centred psychosocial care for patients with CHD, as recommendated by the AEPC psychosocial working group (49).

Our results also suggested that young children with the more severe CHD had significantly poorer HRQoL, as reported by themselves and their parents. In previous studies, disease severity has been found to impact the HRQoL of children with CHD (43). Indeed, HRQoL is significantly impaired in children with hypoplastic left heart syndrome, which is considered as the most severe CHD (50). In their study, Dempster et al. related HRQoL to the levels of impaired adaptive behavior, behavioral symptoms, and functional status (50). Similarly, HRQoL in children aged 2 to 18 y.o. after palliative single ventricle surgical repair, remains lower than in CHD eligible to biventricular surgical repair (44), such as tetralogy of Fallot (34). As in our previous studies in older children, this study confirms the negative impact of repeated invasive cardiac procedures (surgery or catheterization) on HRQoL (10, 51). Moreover, children with a CHD may suffer from an impaired physical capacity, which directly impacts the physical dimension of their HRQoL (6). Some children with CHD may develop an unpleasant feeling of dyspnoea, related to muscular deconditioning or restrictive lung function, which disrupts their physical well-being (22). Most of those children remain eligible to all sports, therefore healthcare providers, families and teachers should contribute to physical activity promotion in this population from an early age, to avoid the vicious deconditioning circle (37). Children with complex CHD may also present with an impaired HRQoL, as they are at higher risk of neurological or behavioural problems, including attention disorders, than children with a simple CHD (44).

In our study, HRQoL was predicted by the disease severity based on the infant heart failure classification from Ross et al. (29). The Ross classification may therefore be more discriminating than the Bethesda classification (30) or the foetal prognosis classification from Davey et al. (31). In the adult population, the strong correlation between NYHA functional status and HRQoL has been widely reported, in chronic heart failure (52), CHD (53) or pulmonary arterial hypertension (18). The Ross classification is mostly used in paediatric drug trials, therefore our results suggest using this scale in routine follow-up, to assess both functional status and HRQoL. We also suggest using self-reported HRQoL outcomes in paediatric cardiology trials.

### Study limitation

HRQoL in this cohort of children with CHD was good and similar to controls but patients were recruited from outpatient visits in expert centres. Therefore, severe conditions have been underestimated (hospitalization, other severe comorbidities, Ross class III and IV), as well non-severe conditions (no follow-up in a tertiary care centre). However, we previously reported HRQoL data on young children with severe conditions (21) and most paediatric cardiologists in our region have been involved in the study.

Many patients with a CHD may also be concerned with a genetic syndrome or a neurological deficit, with a potential impact on their HRQoL. They have purposely not been included in this study for methodological issues (inability to understand and/or fill-in the questionnaires). However, investigating HRQoL in this specific population using appropriate measurement tools remains necessary.

Currently, nearly half CHD are diagnosed prenatally, most future children are expected to benefit from an efficient treatment with a low morbidity and mortality. As a result, many parents ask the following question during prenatal diagnosis: “what will be the quality of life of my future child?”. Therefore, future research, using large international registries, should investigate the reliability of prenatal prognosis assessment, such as the scale from Davey et al., in terms of HRQoL prediction.

## Conclusion

HRQoL in young children with CHD aged 5 to 7 years old was good and similar to controls. HRQoL in this population correlated with CHD severity, especially when assessed by the infant heart failure Ross classification. This study contributed to the growing body of knowledge on HRQoL in congenital cardiology and emphasized the need for child and family support in the most complex CHD. In the current era, prognosis in CHD is excellent, therefore the beneficial role of HRQoL assessment in routine clinical practice for screening and decision-making is of great importance. However, other domains remain under-investigated in the CHD population, such as the impact of psychomotor and cognitive developmental disorders on HRQoL.

## Supplementary information

**Additional file 1:** **Supplementary Table 1.** Health-related quality of life in children with CHD compared to control children. **Supplementary Table 2.** Mother-report QoL explanatory variables in the CHD group. **Supplementary Table 3.** Father-report QoL explanatory variables in the CHD group.

## Data Availability

All data generated or analyzed during this study are included in this published article. The datasets used for those published data are available from the corresponding author on reasonable request.

## Abbreviations

ACC-CHD: Anatomic and clinical classification of congenital heart disease
CHD: Congenital heart disease
HRQoL: Health-related quality of life
NYHA: New York Heart Association Functional Classification
PedsQL: Paediatric Quality of Life Inventory
SD: Standard deviation
SE: Standard error
