# Supplementary material for: Health-related quality of life in children with congenital heart disease aged 5 to 7 years: a multicentre controlled cross-sectional study
Source: Health Qual Life Outcomes. 2020 Nov 12;18:366. doi: 10.1186/s12955-020-01615-6 (PMC7659069; doi:10.1186/s12955-020-01615-6)
Supplement: Supplementary file 1 — Additional file 1: Supplementary Table 1. Health-related quality of life in children with CHD compared to control children. Supplementary Table 2. Mother-report QoL explanatory variables in the CHD group. Supplementary Table 3. Father-report QoL explanatory variables in the CHD group. [file 12955_2020_1615_MOESM1_ESM.docx]

Supplementary Table 1: Health-related quality of life in children with CHD compared to control children

| HRQoL dimension | CHD group scores (means ± SEM) | | Controls group scores (means ± SEM) | | P-value |
| --- | --- | --- | --- | --- | --- |
| **Self-reported HRQoL** | | | | | |
| Physical functioning | | 76.2 ± 1.4 | | 77.4 ± 1.4 | 0.55 |
| Emotional functioning | | 69.1 ± 1.8 | | 67.2 ± 1.8 | 0.47 |
| Social functioning | | 75.2 ± 1.7 | | 74.1 ± 1.7 | 0.65 |
| School functioning | | 72.2 ± 1.6 | | 69.9 ± 1.6 | 0.31 |
| Psychosocial functioning | | 72.1 ± 1.3 | | 70.4 ± 1.3 | 0.36 |
| Total scale score | | 73.5 ± 1.2 | | 72.8 ± 1.2 | 0.68 |
| **Mother-reported HRQoL** | | | | | |
| Physical functioning | | 82.6 ± 1.5 | | 87.1 ± 1.4 | **0.03** |
| Emotional functioning | | 64.7 ± 1.6 | | 67.6 ± 1.6 | 0.21 |
| Social functioning | | 80.5 ± 1.5 | | 86.0 ± 1.5 | **0.01** |
| School functioning | | 73.3 ± 1.6 | | 79.7 ± 1.5 | **<0.01** |
| Psychosocial functioning | | 72.9 ± 1.2 | | 77.8 ± 1.2 | **<0.01** |
| Total scale score | | 76.1 ± 1.1 | | 81.1 ± 1.1 | **<0.01** |
| **Father-reported HRQoL** | | | | | |
| Physical functioning | | 83.3 ± 1.8 | | 89.5 ± 1.5 | **<0.01** |
| Emotional functioning | | 69.8 ± 1.7 | | 72.1 ± 1.5 | 0.30 |
| Social functioning | | 84.3 ± 1.6 | | 87.6 ± 1.4 | 0.12 |
| School functioning | | 76.8 ± 1.8 | | 82.2 ± 1.5 | **0.02** |
| Psychosocial functioning | | 76.9 ± 1.3 | | 80.7 ± 1.1 | **0.03** |
| Total scale score | | 79.2 ± 1.2 | | 83.7 ± 1.1 | **0.01** |

Legend: Values are age and gender-adjusted means (± SEM). Significant p-values < 0.05 are marked in bold.

Supplementary Table 2. Mother-report QoL explanatory variables in the CHD group

|  |  | Physical functioning | | | Psychosocial functioning | | | Total scale score | | | |
| --- | --- | --- | --- | --- | --- | --- | --- | --- | --- | --- | --- |
|  |  | Univariate analysis | | Multivariate analysis | Univariate analysis | | Multivariate analysis | Univariate analysis | | Multivariate analysis | |
| Variables |  | Estimation  (SE) | p-value | p-value ^$^ | Estimation  (SE) | p-value | p-value ^$^ | Estimation  (SE) | p-value | p-value ^$^ |  |
| Age |  | -2.1 (2.0) | 0.30 * | 0.46 | -1.1 (1.6) | 0.50 * | 0.67 | -1.4 (1.6) | 0.35 * | 0.53 |  |
| Gender | Female vs Male | -3.5 (3.4) | 0.31 * | 0.54 | -4.0 (2.7) | 0.13 * | 0.17 | -4.1 (2.6) | 0.12 * | 0.14 |  |
| Bethesda severity classification | Low vs Severe  Moderate vs Severe | 9.5 (5.3)  9.1 (5.4) | 0.19 * | - | 0.6 (4.3)  -2.8 (4.3) | 0.48 | - | 4.6 (4.1)  2.2 (4.2) | 0.48 | - |  |
| Ross classification | Class I vs Class II | 16.7 (5.9) | **0.01** * | **0.01** | 12.3 (4.6) | **0.01** * | **<0.001** | 13.7 (4.5) | **<0.01** * | **<0.001** |  |
| Age at CHD diagnosis | Postnatal vs Prenatal | 6.5 (3.8) | 0.09 * | - | -0.01 (3.0) | 0.99 | - | 2.6 (3.0) | 0.38 | - |  |
| Cardiac surgery | No vs Yes | 1.4 (3.4) | 0.68 | - | 1.5 (2.7) | 0.56 | - | 1.7 (2.6) | 0.51 | - |  |
| Number of cardiac surgical procedures | 0 vs >=3 | 24.2 (12.7) | **0.02** * | - | 13.7 (10.1) | 0.14 * | **-** | 17.4 (9.8) | **0.02** * | **-** |  |
|  | 1 vs >=3 | 26.3 (12.8) |  |  | 14.1 (10.1) |  |  | 18.4 (9.8) |  |  |  |
|  | 2 vs >=3 | 8.7 (14.0) |  |  | 3.2 (11.3) |  |  | 4.3 (10.7) |  |  |  |
| Cardiac catheter | No vs Yes | 8.1 (3.9) | **0.04** | - | 5.2 (3.1) | 0.09 | - | 6.1 (3.0) | **0.05** | - |  |
| Number of cardiac catheter procedures | 0 vs 2 | -3.4 (9.2) | **0.05** * | - | -2.8 (7.2) | 0.11 * | - | -3.2 (7.0) | **0.05** * |  |  |
|  | 1 vs 2 | -13.4 (9.7) |  |  | -9.4 (7.6) |  |  | -10.8 (7.4) |  |  |  |
| Cardiac medication | No vs Yes | 21.9 (5.0) | **<0.001** * | **<0.001** | 11.6 (4.1) | **0.01** * | **0.01** | 15.1 (3.9) | **<0.001** * | **<0.001** |  |
| PAH | No vs Yes | 1.8 (9.4) | 0.84 | - | -0.4 (7.3) | 0.95 | - | 0.2 (7.2) | 0.97 | - |  |
| Normal left ventricle ejection fraction | No vs Yes | 2.8 (7.7) | 0.71 | - | 10.2 (5.7) | 0.08 * | - | 7.6 (5.7) | 0.19 * | - |  |

Legend: CHD, congenital heart disease; PAH, pulmonary arterial hypertension; SD, standard error. Values are linear regression coefficients β (Standard Error) and p-values. Significant p-values are marked in bold. * Candidate variables for multivariate analysis. ^$^: “-” Not maintained in the model (p >0.10).

Supplementary Table 3. Father-report QoL explanatory variables in the CHD group

|  |  | Physical functioning | | | Psychosocial functioning | | | Total scale score | | | |
| --- | --- | --- | --- | --- | --- | --- | --- | --- | --- | --- | --- |
|  |  | Univariate analysis | | Multivariate analysis | Univariate analysis | | Multivariate analysis | Univariate analysis | | Multivariate analysis | |
| Variables |  | Estimation  (SE) | p-value | p-value ^$^ | Estimation  (SE) | p-value | p-value ^$^ | Estimation  (SE) | p-value | p-value ^$^ |  |
| Age |  | -3.9 (2.5) | 0.12 * | 0.07 | -2.0 (1.6) | 0.23 * | 0.12 | -2.7 (1.7) | 0.12 * | **0.05** |  |
| Gender | Female vs Male | -0.4 (4.4) | 0.94 * | 0.14 | 2.5 (2.8) | 0.38 * | 0.74 | 1.4 (2.9) | 0.64 * | 0.41 |  |
| Bethesda severity classification | Low vs Severe  Moderate vs Severe | 3.6 (6.4)  4.6 (6.6) | 0.78 | - | 3.2 (4.1)  2.5 (4.2) | 0.74 | - | 3.4 (4.3)  3.2 (4.4) | 0.71 | - |  |
| Ross classification | Class I vs Class II | 5.1 (8.2) | 0.53 | **-** | -1.6 (5.2) | 0.76 | - | 0.8 (5.4) | 0.89 | - |  |
| Age at CHD diagnosis | Postnatal vs Prenatal | 5.8 (4.8) | 0.23 | - | -1.0 (3.2) | 0.75 | - | 1.3 (3.2) | 0.69 | - |  |
| Cardiac surgery | No vs Yes | 6.7 (4.2) | 0.12 | - | 5.7 (2.7) | **0.03** | - | 6.0 (2.7) | **0.03** | - |  |
| Number of cardiac surgical procedures | 0 vs 2 | 13.1 (8.1) | 0.19 * | - | 12.7 (5.1) | **0.03** * | - | 12.9 (5.2) | **0.03** | - |  |
|  | 1 vs 2 | 7.6 (8.3) |  |  | 8.4 (5.2) |  |  | 8.2 (5.3) |  |  |  |
| Cardiac catheter | No vs Yes | -2.4 (4.9) | 0.63 | - | 0.9 (3.1) | 0.76 | - | -0.2 (3.2) | 0.95 | - |  |
| Number of cardiac catheter procedures | 0 vs 2 | -13.0 (9.7) | 0.40 | - | -3.5 (6.3) | 0.68 | - | -6.9 (6.4) | 0.49 | - |  |
|  | 1 vs 2 | -13.3 (10.5) |  |  | -5.6 (6.8) |  |  | -8.3 (6.9) |  |  |  |
| Cardiac medication | No vs Yes | 2.3 (8.2) | 0.78 | **-** | 6.5 (5.2) | 0.22 | - | 5.0 (5.4) | 0.36 | - |  |
| PAH | No vs Yes | -8.0 (11.3) | 0.48 | - | -9.6 (7.2) | 0.18 * | - | -9.0 (7.5) | 0.23 | - |  |
| Normal left ventricle ejection fraction | No vs Yes | 7.1 (8.3) | 0.39 | - | 8.9 (5.6) | 0.12 * | - | 8.2 (5.6) | 0.15 | - |  |

Legend: CHD, congenital heart disease; PAH, pulmonary arterial hypertension; SD, standard error. Values are linear regression coefficients β (Standard Error) and p-values. Significant p-values are marked in bold. * Candidate variables for multivariate analysis. ^$^: “-” Not maintained in the model (p >0.10).
